# Speed and Lateral Inhibition of Stimulus Processing Contribute to Individual Differences in Stroop-Task Performance

**DOI:** 10.3389/fpsyg.2016.00822

**Published:** 2016-06-01

**Authors:** Marnix Naber, Anneke Vedder, Stephen B. R. E. Brown, Sander Nieuwenhuis

**Affiliations:** ^1^Experimental Psychology, Utrecht UniversityUtrecht, Netherlands; ^2^Vision Sciences Laboratory, Harvard University, CambridgeMA, USA; ^3^Cognitive Psychology, Leiden UniversityLeiden, Netherlands; ^4^Leiden Institute for Brain and Cognition, Leiden University Medical CenterLeiden, Netherlands; ^5^Clinical Psychology, Utrecht UniversityUtrecht, Netherlands

**Keywords:** Stroop, interference, executive control, motion-induced blindness, P3

## Abstract

The Stroop task is a popular neuropsychological test that measures executive control. Strong Stroop interference is commonly interpreted in neuropsychology as a diagnostic marker of impairment in executive control, possibly reflecting executive dysfunction. However, popular models of the Stroop task indicate that several other aspects of color and word processing may also account for individual differences in the Stroop task, independent of executive control. Here we use new approaches to investigate the degree to which individual differences in Stroop interference correlate with the relative processing speed of word and color stimuli, and the lateral inhibition between visual stimuli. We conducted an electrophysiological and behavioral experiment to measure (1) how quickly an individual’s brain processes words and colors presented in isolation (P3 latency), and (2) the strength of an individual’s lateral inhibition between visual representations with a visual illusion. Both measures explained at least 40% of the variance in Stroop interference across individuals. As these measures were obtained in contexts not requiring any executive control, we conclude that the Stroop effect also measures an individual’s pre-set way of processing visual features such as words and colors. This study highlights the important contributions of stimulus processing speed and lateral inhibition to individual differences in Stroop interference, and challenges the general view that the Stroop task primarily assesses executive control.

## Introduction

Because of cognitive processing limitations, observers can only attend a limited set of objects in their surroundings at a time (Broadbent, 1958; Neisser, 1967; Schneider and Shiffrin, 1977; Tsotsos, 1990; Verghese and Pelli, 1992; Rensink et al., 1997). This limit is inherent to an ongoing competition between stimuli in our sensory perception. Depending on its properties, being either weak (e.g., a low-contrast visual stimulus) or strong (e.g., a colorful display), a stimulus may automatically attract more processing resources than others (Treisman, 1969; Eriksen and Eriksen, 1974; Duncan, 1984), while other stimuli need voluntary effort to be processed accurately

(Posner and Snyder, 1975; Schneider and Shiffrin, 1977). Executive control is a term often used to describe an additional function that allows an individual to selectively attend to a desired object and ignore other features (Cohen et al., 1990; Desimone and Duncan, 1995; Miller and Cohen, 2001). Individual differences in these mechanisms may explain, for example, why some can read a book in a noisy café or train without becoming distracted, while others can barely think clearly under such circumstances. A deficit in the tendency to be distracted or the ability to control attention, either because of genetic predisposition, aging, brain disease, or fatigue, has a devastating impact on a person’s cognition (Sarter and Paolone, 2011). Hence, it is important to detect and measure such impairments accurately in practice.

### Factors Determining Individual Differences in Attention

**FIGURE 1:**
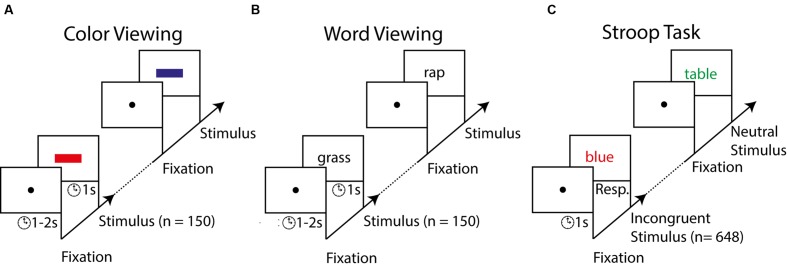
**Stimuli and procedure of Experiment 1.** Participants first passively viewed, in separate blocks, series of colored rectangles and colorless (i.e., black) words **(A,B)**. Participants subsequently carried out the Stroop task in which they had to report the font color of neutral (e.g., table) or color words (e.g., blue) as fast as possible **(C)**. Color words were incongruent or congruent with the font color.

The color-word Stroop task (Stroop, 1935) is a popular (neuro-) psychological method in experimental and clinical practice. The task generally used to measure a person’s executive control and selective attention (MacLeod, 1991, 1992) as a reflection of frontal-lobe functioning (Perret, 1974). **Figure 1C** shows an example of an incongruent (“BLUE”) and neutral word (“TABLE”) in a computerized version of the Stroop task. A subject is required to report the printed ink color of each word as fast as possible. This is difficult when the color of the word is incongruent with its meaning because the word content distracts and interferes with the subject’s report of the ink color. The degree of Stroop interference is a believed to be a reflection of voluntary control (Smith and Kosslyn, 2007), and the degree of interference in an individual is commonly used as a measure of executive control in a range of neuropsychological patient populations (Fisher et al., 1990; Hänninen et al., 1997; McGrath et al., 1997; MacLeod and MacDonald, 2000; Salo et al., 2001). While executive control is a rather broad term that is often used to describe a set of multiple skills (Miyake et al., 2000), in the context of the Stroop task it specifically refers to the active maintenance of a task goal representation which biases information processing in favor of the task-relevant stimulus-response mappings (Cohen et al., 1990; Miller and Cohen, 2001). Stroop-task interference scores thus are thought to measure to what degree a person can execute top-down control and selectively bias attentional deployment of resources to the relevant feature (Kerns et al., 2004).

However, popular models of the Stroop task suggest that individual differences in Stroop interference rely on several other factors (Cohen et al., 1990, 1992; MacLeod, 1991; MacLeod and MacDonald, 2000; Roelofs, 2003). The parallel processing of words and colors via distinct pathways gives way to several additional factors that may account for individual differences in Stroop interference. For instance, people may vary in their proficiency to process words, a factor that may influence the amount and timing of distraction by word meaning while doing the Stroop task (Protopapas et al., 2007). Poor readers may need more time to understand word content, which may increase attention to words, resulting in stronger distraction and therewith more interference in the Stroop task. The mutual inhibition between the visual word- and color-processing pathways is another potential factor that could affect the degree of Stroop interference (Cohen and Huston, 1994). People may vary in the strength of lateral inhibition between the word and color features at one or more levels of representations. Here, we sought to determine to what degree individual differences in Stroop interference are related to the distinct neural pathways of color and word processing and the lateral inhibition between visual feature representations.

### Speed Discrepancies in Parallel Stimulus Processing

There is considerable evidence that the instigation of interference requires a relative contrast in the automaticity in the processing of each stimulus (Cattell, 1886; Fraisse, 1969; Dyer, 1973; MacLeod and Dunbar, 1988). In the Stroop task reading word content is more “automatic” and therefore receives priority in processing over the less exercised behavior of color naming. Evidence for this comes from *between-group* studies that tested practice effects on interference. For example, poor readers report colors faster than words and have less interference while good (i.e., practiced) readers show the opposite pattern (Corbitt, 1978). Furthermore, Stroop studies on bilinguals show that interference is stronger when words are presented to bilinguals in their highly practiced, first language (Preston and Lambert, 1969; Dyer, 1971a), suggesting that bilinguals are distracted more by words when they are proficient in reading. However, counterintuitive evidence comes from studies with children: children with poor reading skills experience similar (Alwitt, 1966) or even more Stroop interference (Everatt et al., 1997; Protopapas et al., 2007), which could reflect an increase in distraction by words due to increased attentional effort to process them. Although these studies demonstrate the importance of practice in sensory processing of a specific feature modality, it remains unknown how much variance in interference across individuals *within* a homogeneous group is explained by the difference in processing speed of words versus colors. Previous studies were unable to disentangle processing speed from the amount of effort allocated to resolve a perceptual conflict. Simply put, no conflict can occur and no attentional control is necessary when the word in the Stroop task is not processed before the color. As is the case with illiterates and children that still have to learn to read, word information is processed too late to interfere with color-naming in the Stroop task. This is congruent with the idea that interference reflects delays in processing stages preceding responses to colour (Dyer, 1971b; Glaser and Glaser, 1982; Lansbergen and Kenemans, 2008). On the other hand, slow reading of words can also reflect an increase in attentional resources to word content, resulting in more distraction and stronger interference in the Stroop task. Hence, the separate contributions to Stroop interference by either attentional control or relative processing speed between the conflicting color and word stimuli have remained elusive. Here we describe a novel approach to resolve this problem by measuring to what extent relative parallel sensory processing determines interference scores, independent of executive control.

### Interaction and Competition between Parallel Stimulus Processing Pathways

A second factor that may determine the degree of Stroop interference is the interaction between the two parallel pathways responsible for the processing of colors and words. Interference in the Stroop task is characterized by a competition between the color and word feature representations. There is consistent evidence that the anterior cingulate cortex plays a role in monitoring or resolving such competition at the response level during the Stroop task (Pardo et al., 1990; MacLeod and MacDonald, 2000; Barch et al., 2001; Botvinick et al., 2004). Conflict in the Stroop task can additionally be the result of the overlap between sensory representations of words and colors. At some processing stage, the interfering, irrelevant word meaning appeals to similar resources and competes to overrule the relevant color stimulus (Logan et al., 1984; Cohen et al., 1990; Phaf et al., 1990). Very similar to perceptual suppression in which stimuli compete – to a degree that they can even render each other invisible (Naber et al., 2009) –, words and colors also mutually inhibit each other in the Stroop task as they compete for processing resources. There is, however, no direct evidence that varying degrees of inhibition underlie individual differences in Stroop-task performance within a subject population. This is because it is difficult to determine the influence of lateral inhibition on Stroop interference independent of executive control because inhibition is a prerequisite of executive control: when stimuli do not compete perceptually (e.g., in proximity), there is no conflict and nothing to control and resolve.

### Disentangling Processing Speed and Inhibition from Executive Control

A solution to the problem of entanglement of interference, processing speed, and inhibition, is to measure the processes independently and correlate their influence across individuals. Independent of response conflict and executive control, people may have a brain system that is predisposed to process words relatively slowly as compared to colors, and is equipped with relatively strong lateral inhibition between features. While there is some evidence to support links between interference, speed of stimulus processing and mutual competition between perceptual representations of stimuli, so far no studies have examined the weight of these relations. Using a correlational approach, we explore to what extent the relative processing speed of words versus colors (Experiment 1) and lateral inhibition between visual features (Experiment 2) explain individual variability in interference.

## Experiments

### Experiment 1

Experiment 1 was designed to demonstrate the role of the relative difference in the speed of processing words and colors in determining the size of Stroop interference. To measure the effects of processing speed per feature while circumventing the influence of conflict monitoring and resolution, we measured event-related potentials (ERPs) while participants passively watched a series of color and word stimuli presented in isolation. The peak latencies of the P3 components associated with color and word processing were taken as an index of the speed of evaluation of these stimuli (Kutas et al., 1977; McCarthy and Donchin, 1981). This assumption is supported by evidence that P3 amplitude cumulatively rises as more perceptual evidence is gathered, reaching its peak when sufficient visual information has been processed to form a decision about the identity of the stimulus (McCarthy and Donchin, 1981; O’Connell et al., 2012; Twomey et al., 2015). The passive P3 is also known as a measure of verbal fluency (O’Donnell et al., 1992). After the ERP measurements, participants performed a typical Stroop task and we determined the behavioral Stroop interference scores. The main goal was to examine if the size of an individual’s Stroop interference score was related to the relative latencies of the P3 components elicited by isolated features.

#### Material and Methods

##### Participants

Twenty-six Dutch students (age range: 18–51, 14 females) were recruited for the EEG experiment through an online research participation system (Sona systems, Tallinn, Estonia). Participation was only possible for participants who were healthy, right-handed, non-color blind, non-dyslectic, and native Dutch speakers. All these factors were self-assessed by the participants before they signed up but color blindness was double-checked with Ishihara plates in the laboratory right before participation. Three participants were unable to complete the experiment due to technical difficulties. All participants had normal or corrected-to-normal vision, were naive to the purpose of the experiment, and gave written informed consent before the experiment. The experiments conformed to the ethical principles of the Declaration of Helsinki and were approved by a local ethics committee of the University. Participants’ either received course credit or money (15 euro) for participation.

##### Stimuli and materials

The stimuli consisted of colored rectangles and Dutch words presented in sequences on a black screen preceded by a white fixation dot (**Figure 1**). The rectangles were 30 by 60 pixels and presented in the color red, green, or blue. Words were chosen from a list of 50 Dutch words that were relatively similar in phonology and morphology to the color words “red,” “blue,” and “green,” matched in word frequency and word length, and presented in Helvetica font size 36. The fixation dot was 10 pixels in diameter. Stimuli were presented on a 21-inch Iiyama CRT monitor (Iiyama, Tokyo, Japan). Viewing distance to the screen was approximately 75 cm. The refresh rate of the screen was 85 Hz and the resolution was 1600 by 1200 pixels. Stimuli were generated on a Dell computer (Dell, Round Rock, TX, USA), using Matlab (Mathworks, Natick, MA, USA) and the Psychtoolbox extension.

##### Procedures

*Color and word viewing task.* We designed the EEG experiment to examine to what degree the brain’s responses to colors and words predict Stroop interference scores. As a reflection of a person’s speed of processing such individual features, we measured P3 latencies to colored rectangles and colorless words presented in isolation. Participants looked at the presentation of 150 rectangles with a randomly intermixed color (**Figure 1A**). We additionally presented 150 white, colorless words with a content randomly chosen from the list of 50 words (**Figure 1B**). Participants were instructed to carefully inspect each stimulus but that no immediate response was required. Each stimulus was depicted for 1 s and a blank black screen with a fixation point preceded each stimulus. To circumvent potential brain responses associated with stimulus anticipation, the blank screens were presented for a duration randomly selected from a uniform distribution between 1 and 2 s. The order of the color block and word block was counterbalanced across participants. The participants took a 10-s break after every sequence of 60 stimulus presentations.

*Stroop task*. Next, the participants performed a typical computerized version of the Stroop task (**Figure 1C**). We used a computerized version because of its many advantages over the verbal Stroop task (Salo et al., 2001). The most relevant advantages are that a computer version of the Stroop task requires no verbal pronunciation of the ink colors and includes randomized conditions across trials. In the verbal Stroop task reading speed is taken into account by contrasting speed of reading colorless words, naming colors, and reading incongruent words (Golden, 1978). However, the standard method of correcting for reading skills has been criticized for its validity (Lansbergen et al., 2007a). Nevertheless, the computerized (manual) version of the Stroop task produces comparable Stroop effects and ERP responses as the verbal Stroop task (Liotti et al., 2000). In our version of the task, participants had to respond with rapid button presses to Dutch words presented on a computer screen (Dalrymple-Alford and Budayr, 1966; Logan et al., 1984). Participants were shown 216 incongruent, 216 congruent, and 216 neutral words in random order. We added the congruent condition to lower the relative frequency of incongruent trials, which results in stronger Stroop interference (Logan and Zbrodoff, 1979; Tzelgov et al., 1992; Lansbergen et al., 2007b). On incongruent trials, a word describing a color was presented in a mismatching font color (e.g., “RED”). On congruent trials, the word content matched its font color (e.g., “RED”). On neutral trials, the colored words did not refer to a color (e.g., “TABLE” or “CHAIR”). Word presentations were preceded by a fixation screen for 1 s and as soon as the word was shown, participants were required to report the word’s font color as fast as possible. Trials in which participants did not respond within 1 s or made the wrong answer were removed from the analysis. Participants reported a word’s font color by pressing one of the three arrow keys (left, down, and right) on a keyboard. The word disappeared when a response was registered and was followed by the reappearance of the fixation dot. Feedback was only given in the first 18 trials by changing the color of the fixation dot for 500 ms (red = incorrect response, green = correct, and blue = too late). Participants were asked to minimize blinks and eye-movements, and maintain steady fixation centered at the words. The participants took a 10-s break every 60 trials and a long break of 3 min halfway through the Stroop task.

##### EEG acquisition and analysis

**FIGURE 2:**
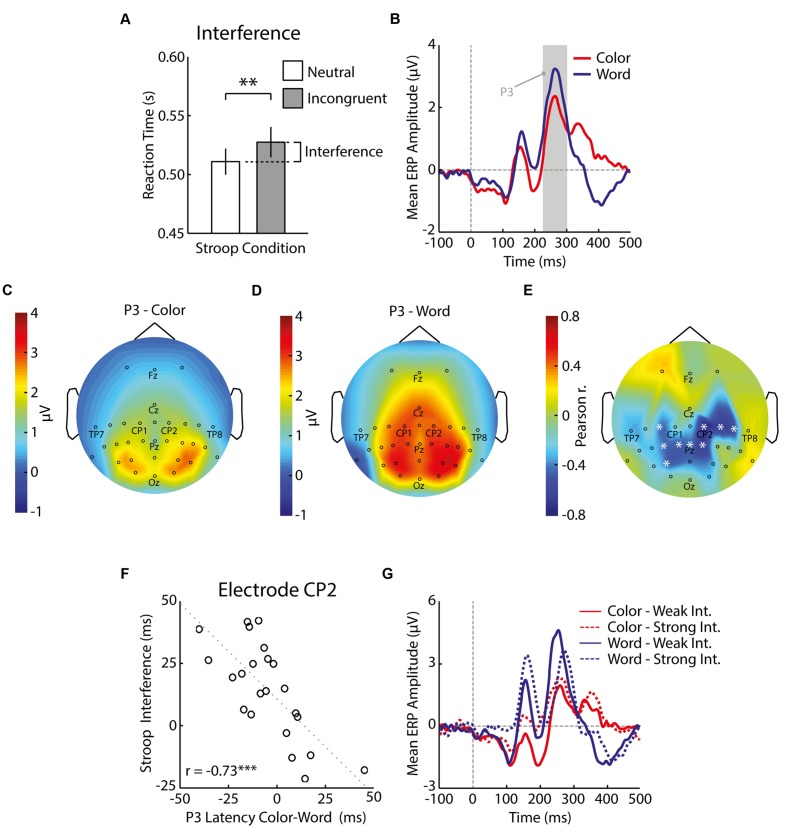
**Behavioral and electrophysiological results of Experiment 1.** The bar graph with mean and standard error of reaction times shows that incongruent trials induced significantly slower responses (^∗∗^*p* < 0.01) than neutral trials **(A)**. Grand-average ERPs (i.e., average across all electrodes and subsequently across all participants) depict brain responses to colored rectangles (red) and colorless words (blue) presented in isolation **(B)**. The gray patch shows the period of the P3 (250–300 ms) and the dashed vertical gray line indicates the time of stimulus presentation. Scalp distributions of the P3 to colors **(C)** and words **(D)** reflect the average EEG signal per electrode (data was averaged in the window specified above per participant and subsequently averaged across all participants). The relative P3 latency of colors minus words correlated with Stroop interference, especially over parietal and right central-parietal regions **(E)**, and the correlation was highest at CP2 **(F)**. Each electrode that is replaced by a white asterisk in **(E)** indicates a significant correlation (*p* < 0.05), and each circle in **(F)** indicates the average difference in reaction times between incongruent and neutral Stroop trials and average difference in P3 latency to color and word stimuli per participant (^∗∗∗^*p* < 0.001). The dotted line indicates a linear regression line fitted to the data. The negative correlations imply that participants whose word P3 peaked later relative to the color P3 showed stronger Stroop interference **(G)**. The legend indicates ERPs to colors (red) and words (blue) for participants with either weak (solid lines) or strong (dashed lines) interference in the Stroop task, as determined by a median split.

Electrophysiological data were recorded with 32 active Bio-Semi electrodes. Most electrodes were placed over posterior brain areas, where the classic P3 is largest in amplitude (see **Figures 2C–E**). We placed the reference electrodes at the mastoids and four additional electrodes around the eyes (below and above the right eye, left of the left eye, and right of the right eye) to detect blinks and eye-movement artifacts. The data were recorded at 1024 samples per second in the program ActiView and analyzed in MatLab using the FieldTrip (Oostenveld et al., 2011) and EEGLAB (Delorme and Makeig, 2004) toolboxes. During acquisition, impedances were kept below 30 kΩ. Each electrode was measured on-line with respect to a common mode sense active electrode producing a monopolar channel. EEG data were re-referenced to the average of the left and right mastoid electrodes. Next, data were epoched from 2 s before until 3 s after stimulus onset, high-pass filtered at 1 Hz and low-pass filtered at 60 Hz with a conventional Butterworth filter, and baseline-corrected to the 200-ms interval preceding stimulus onset. Ocular and eye-blink artifacts were detected and removed using the independent component analysis method implemented in FieldTrip. Epochs with excessive signal variance due to transient artifacts were discarded (*M* = 5% of the trials, *SD* = 3%). Stimulus-locked ERP waveforms were obtained by averaging epochs separately for color and word stimuli. P3 latency was defined as the last positive ERP peak between 200 and 300 ms after stimulus onset.

*Behavioral reaction time analysis.* As is common in Stroop-task research, interference scores were defined as the difference in median reaction time for correct incongruent trials and correct neutral trials. A positive difference indicated that participants experienced strong interference from the distracting word content. We do not report the facilitatory effect of congruent trials because we found no relationship between the size of this effect and the difference between words and colors in ERP component latencies/amplitudes. Moreover, it is difficult to verify whether participants attended to the word or the font color in congruent trials (Spieler et al., 1996). All reported statistical comparisons between stimulus conditions were paired two-sided *t*-tests.

#### Results and Discussion

We first checked whether participants made more errors and responded more slowly in incongruent trials than neutral trials. As expected, average response accuracy was significantly lower for incongruent than for neutral trials [incongruent: 87% ± 7%, neutral: 90% ± 7%, *t*(22) = 3.38, *p* = 0.003, Cohen’s *d*_z_ = 0.70, Cohen’s *d*_rm_ = 0.42]. Correct reactions in incongruent trials were 15 ms slower than correct reactions in neutral trials [incongruent: 520 ± 5 ms, neutral: 505 ± 6 ms, *t*(22) = 3.60, *p* = 0.002, Cohen’s *d*_z_ = 0.75, Cohen’s *d*_rm_ = 0.49; **Figure 2A**]. To calculate split-half reliability of Stroop interference, we correlated the interference scores for odd-numbered and even-numbered trials across participants, and adjusted the correlation using the Spearman–Brown prediction formula. This analysis indicated a high reliability of Stroop interferences scores: 0.99. In sum, we found a Stroop effect for incongruent words in most individuals.

The grand-average ERPs elicited by the color and word stimuli presented during the preceding passive viewing task showed distinct P3 components (**Figure 2B**), with an occipital-parietal scalp distribution for colors and a central-parietal distribution for words (**Figures 2C,D**). We found that individual variability in the relative latency of the color- and word-related P3s predicted Stroop interference scores (**Figures 2E,F**; for correlations, see Supplemental Materials): Participants whose word-related P3 peaked late relative to their color-related P3 experienced increased Stroop interference (test–retest reliability of relative latencies of P3 at CP2: 0.84). **Figure 2G** shows word- and color-related ERP waveforms from electrode CP2 for individuals with weak (*n* = 11) and strong (*n* = 12) Stroop interference (based on median split). The groups differed in the latency of the word-related P3 [weak interference: 252 ms ± 10 ms, strong interference: 271 ms ± 16 ms, *t*(21) = 3.43, *p* = 0.003, Cohen’s *d*_s_ = 1.43], while the peak latency of their color-related P3 was essentially the same [weak interference: 260 ms ± 13 ms, strong interference: 256 ms ± 21 ms, *t*(21) = 0.59, *p* = 0.562, Cohen’s *d*_s_ = 0.25]. P3 amplitude did not significantly differ between groups (*p’s* > 0.05).

In sum, these results suggest that a delay in word processing relative to color processing, as reflected in a later P3 component, predicts strong Stroop interference in a homogenous group of highly educated young adults. This finding may appear counterintuitive because a delayed word processing could, hypothetically, result in a situation where word meaning is processed too late to interfere with color naming, hence causing weak Stroop interference. In the light of the current results, however, it is more likely that the delay in word processing is accompanied with a demand for increased attentional focus for accurate word processing, therewith inducing even more distraction by word meaning. This interpretation is in line with work by Protopapas et al. (2007) who showed that a heterogeneous group of schoolchildren with reading difficulties had increased Stroop interference.

An alternative explanation is that the word reading process has to be completed before a response to colors can be made. This interpretation is in line with Roelofs (2003) view on the Stroop task, according to which interference is directly related to the time it takes for a reader to discover that the concept (lemma) of the written word in the Stroop task is not consistent with the color-naming goal. This suggests that the duration of the activation of the wrong concept and consecutive blocking of the correct concept depends on how fast people can encode words. This assumption is supported by the observation that uncommon words that are more difficult to encode cause slower color-naming response times in incongruent trials of the Stroop task (Burt, 2002).

Although this needs further exploration, the P3 latency as measured with EEG may reflect the accumulation of evidence that is needed to decide what the word meaning is and whether it needs to be inhibited. It is important to note that we measured the P3s elicited by words and colors presented in isolation and in the absence of an executive control task. We therefore speculate that the word- and color-related P3 latencies are a sensitive index of the natural processing speed of the word-reading and color-naming pathways that are usually brought in competition during the Stroop task. Individuals with a processing pathway characterized by greater natural processing speed of words may have an earlier P3 peak for word stimuli (and less interference). In contrast, late P3 peaks could reflect slower word reading, leading to strong interference.

### Experiment 2a

The second relevant factor that may determine Stroop interference scores is the degree of lateral inhibition: the extent to which the neural representations of the color stimulus and the word stimulus mutually inhibit each other (Cohen et al., 1992; Usher and McClelland, 2001). In popular Stroop models the inhibitory interaction between color and words plays an essential role at several stages along the hierarchy of the processing of incongruent Stroop stimuli (Cohen and Huston, 1994; Botvinick et al., 2001). Independent of the activation of more executive control to quickly respond to incongruent stimuli and independent of relative stimulus processing speed, as reported above, an individual may have a pre-set degree of lateral inhibition between stimulus features. Such pre-set global processing styles have recently been shown to affect interference in conflict tasks (Shin and Kim, 2015). The question is how we can measure to what degree individuals are sensitive to lateral inhibition between features?

**FIGURE 3:**
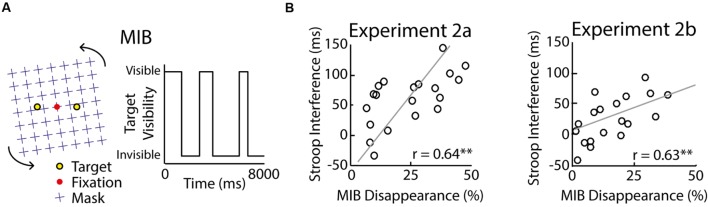
**Results of Experiment 2.** In the MIB illusion a rotating mask (left, blue) induces the disappearance of perceptual targets (left, yellow) for variable durations (right) depending on the amount of target inhibition by the mask **(A)**. Participants have to fixate the red dot in the center to experience MIB. Stroop-task interference correlated significantly with MIB strength in Experiment 2a (**B**; left) and Experiment 2b (**B**; right). Significance of the correlations is indicated with an asterisk (^∗∗^*p* < 0.01). The raw correlation is depicted, not the partial correlation.

A task known to measure the strength of lateral inhibition between perceptual features is the report of illusory target disappearances in motion-induced blindness (MIB) (Bonneh et al., 2001; van Loon et al., 2013). The illusion consists of the perceptual disappearance of peripheral (and therefore weak) targets when displayed upon a stronger central motion mask (**Figure 3A**). Bonneh discovered MIB and proposed that peripheral targets disappear from consciousness because they lose the competition with the salient mask due to inhibition. In line with this theory, studies have demonstrated that a weaker target or more salient mask causes more and longer target disappearances (Bonneh et al., 2001; Graf et al., 2002; Wilke et al., 2003; Naber et al., 2009). Lateral inhibition is a key mechanism that drives the distribution of attention to objects and explains a large array of phenomena, including visual search, dual-task performance, and feature discrimination experiments (Treisman, 1969; Eriksen and Eriksen, 1974; Duncan, 1984; Desimone and Duncan, 1995). Other MIB-like phenomena are subject to competition in the form of lateral inhibition as well (Bonneh et al., 2001; Kanai and Kamitani, 2003; Caetta et al., 2007; Kawabe et al., 2007; Naber et al., 2009; Schölvinck and Rees, 2009), and models that simulate changes in conscious content incorporate lateral inhibition as a key parameter to explain individual differences in the duration of percepts (Lehky, 1988; Noest et al., 2007; van Loon et al., 2013). Importantly, the strength of inhibition markedly differs across individuals, with some experiencing much longer disappearances than others, a variable that is thought to be determined by concentrations of the inhibitory neurotransmitter GABA in the occipital lobe (Edden et al., 2009; Yoon et al., 2010; van Loon et al., 2013). We next investigate to what degree individuals vary in MIB and whether this variability correlates with the variability in performance on the Stroop task, which may similarly depend on a pre-set degree of lateral inhibition between perceptual feature representations and their processing pathways.

#### Materials and Methods

##### Participants

Twenty healthy, right-handed, non-color blind, non-dyslectic native-speaking Dutch individuals participated in this experiment. Their age ranged from 20 to 32 years and 10 were male, 10 female. All participants had normal or corrected-to-normal vision, were naive to the purpose of the experiment, and gave written informed consent before the experiment. The experiments conformed to the ethical principles of the Declaration of Helsinki and were in accordance with the ethical guidelines of the University.

##### Stimuli – Motion-Induced Blindness

The MIB illusion consists of peripheral targets that are involuntarily rendered invisible at random moments when observers fixate the centre of a distracting motion stimulus (for a demo, see http://www.michaelbach.de/ot/mot-mib/index.html). Specifically, distracting motion patterns are thought to draw processing resources away from the less salient target, therewith leaving insufficient resources for the stimulus to reach perceptual awareness. As shown in the right panel of **Figure 3A**, observers fixated a red dot that was located at the center of a black background. A distracting clockwise rotating pattern of blue crosses (size: ∼20 × 20°) was shown in the background and *two* yellow stationary targets (diameter: ∼0.5°) were superimposed left and right from the target at the horizontal meridian (eccentricity: ∼5°). Depending on the participant’s perception, the yellow target disappeared irregularly with particular durations.

##### Stimuli – Stroop task

All aspects were similar to Experiment 1 except that we used Dutch words and increased the number of colors from three to four. Words were matched in word frequency and word length across conditions.

##### Materials

All stimuli were presented on a computer with a 24 × 32 cm (∼15′) screen (resolution: 800 × 600 pixels; refresh rate: 75 Hz). The keys used for responding in the Stroop task were color-labeled on the keyboard. Participants had an approximate viewing distance of 50 cm to the screen. The MIB task stimuli were run in MATLAB and Stroop stimuli were presented using E-Prime. The tasks were performed in a dark room.

##### Procedures

Before the experiment participants performed a few practice trials to form an impression of the stimuli and tasks. All participants performed a 5-min MIB task, which was followed by the Stroop task and subsequently another 5-min MIB task. Depending on the participant’s reaction times, the Stroop task lasted approximately 5 min.

*Motion-Induced Blindness task*. The MIB task started with the presentation of the red fixation dot that was fixated by the participants. Two seconds later, the rotating pattern and yellow targets were shown. Participants were instructed to maintain fixation (i.e., minimize eye movements) and simultaneously report the disappearance of targets using the left and right arrow buttons on a keyboard that corresponded to the location of both targets (**Figure 3A**). Participants were told that they had to press the button to report a disappearance onset and hold the button until the target reappeared. Participants were further instructed to only report full disappearances and to ignore target fading (i.e., transparent luminance decreases). Participants were *not* pointed out that the fading and disappearance of targets were visual illusions and a result of cognition.

*Stroop task.* All aspects were similar to Experiment 1 except that this Stroop task consisted of 36 incongruent trials, 36 congruent, and 32 neutral trials, intermixed, presented in random order. Word presentations were preceded by blank gray screens. Participants reported a word’s font color by pressing one of four keys (F1, F2, F11, and F12) on a keyboard that each corresponded to a color (“RED,” “GREEN,” “BLUE,” and “YELLOW”).

##### Analysis

The strength of the MIB illusion was measured as the total percentage of the time that the target disappeared during the experiment. Stroop interference was defined as the difference between the median reaction times on incongruent and neutral trials. All reported statistical comparisons between conditions (*t*-tests) and correlations between Stroop interference scores and MIB measures (Pearson) were two-sided and paired. For ease of terminology, we call the word content in the Stroop task and the blue rotating pattern in the MIB task the *distracters* and the word color in the Stroop task and the stationary yellow dot in the MIB task the *targets*.

#### Results and Discussion

In Experiment 2a, 20 participants performed the Stroop task and a MIB task. We measured their Stroop interference scores and the total percentage of time during which the MIB target was subjectively invisible. A Stroop effect was observed with delayed median reaction times for incongruent as compared to neutral trials [incongruent: *M* = 737 ms, *SD* = 104 ms; neutral: *M* = 678 ms, *SD* = 105 ms; *t*(19) = 6.31, *p* < 0.001]. Accuracy scores matched the pattern of Stroop interference [incongruent: 89% ± 8%, neutral: 93% ± 6%, *t*(19) = 2.71, *p* = 0.014, Cohen’s *d*_z_ = 0.61, Cohen’s *d*_rm_ = 0.53]. As shown in **Figure 3B** (*left*), the Stroop interference scores and MIB strength correlated across participants (Spearman–Brown-adjusted split-half reliability of Stroop interference: 0.64; MIB disappearance: 0.97). These results suggest that Stroop interference and the illusion strength in MIB are related. We propose that both phenomena are linked, possibly through the degree of lateral inhibition between visual representations, which plays an important role in models of the Stroop task (Cohen and Huston, 1994) and theories on MIB (Caetta et al., 2007; Gorea and Caetta, 2009).

### Experiment 2b

In Experiment 2B we attempted to replicate the results in the previous experiment, using somewhat different task parameters, to examine whether the correlation between Stroop interference and MIB is robust to superficial changes in stimulus and task design. In case we find a similar correlation between Stroop interference and MIB, we can conclude that the correlation was caused by commonalities between the tasks. Importantly, in this follow-up experiment participants performed an n-back working-memory task in addition to the Stroop task and MIB task. Adding a third task, allowed us to exclude several potential alternative explanations for the correlation. First, the correlation may reflect fatigue, motivation or any other general construct that can influence performance in both tasks in a systematic fashion (Locke and Latham, 1990; Lorist et al., 2002). If the correlation between Stroop interference and MIB obtained in Experiment 2a reflected individual differences in factors related to the participant’s personal state, Stroop interference and MIB should also correlate with n-back task performance. However, if we replicate the findings in Experiment 2a and find no correlations with n-back task performance, we can conclude with greater confidence that the correlation between Stroop interference and MIB is specific and not due to a general factor. Second, the n-back task is a typical measure of attentional control (Kane et al., 2007) and should correlate with Stroop interference and MIB in case this construct underlies the previously found correlation.

#### Materials and Methods

All aspects of Experiment 2b were similar to Experiment 2a, except for the following. A new sample of 19 American participants was recruited. Their age ranged from 18 to 31 years and 12 were female. The experiment received ethics approval by the international review board of the University.

The MIB rotating distracter stimulus consisted of 200 magenta dots (size: 0.3°) rotating counter clockwise (36°/s) in a circular region with a radius of 13°. The dots positions were randomly allocated in this region at the start of the task. A *single* target was presented at top-right from fixation (at 45° angle from the horizontal and vertical meridians; size: 0.3°; eccentricity: 6.5°) and superimposed on a black circular “security zone” (for details, see Bonneh et al., 2001) that prevented the distracter to directly “touch” the targets (radius: 1.5°).

To verify whether the participant was attending the targets, three catch trials were implemented in the MIB task (at 100, 200, and 300 s after trial onset) by physically removing the target from the screen for a duration that was matched to the median duration of preceding subjective target disappearance durations. The fading of MIB targets in the catch trials were gradual transitions in color saturation (linear fading in or out within 0.1 s) to make the disappearances appear natural. All participants correctly detected each catch trial.

Neutral words in the Stroop task consisted of “END,” “GUESS,” and “BEAM.” The number of trials depended on how many trials a participant could finish within 5 min (∼225 trials). Stimuli were presented on a large Apple screen (40 × 60 cm; 1920 × 1200 pixel; 60 Hz) and the participant’s viewing distance (50 cm) was steadied with a chin-rest.

The working-memory task consisted of a color version of the 2-back task. Participants viewed a sequence of colored dots (size: 0.6°; colors: red, green, blue, cyan, magenta, or yellow), presented one at a time for 1 s, followed by the presentation of a fixation dot for 1 s. Participants were instructed to press the space button as fast as possible (within 1 s) each time the color shown was the same as the color shown 2 presentations before (e.g., “green,” “**red,**” “blue,” “**red**”). The working-memory task lasted 5 min. Feedback about the participant’s responses was only given in the first 30 trials by changing the color of the fixation dot to red, green, or blue for incorrect, correct, or missed trials, respectively. The participant’s total numbers of hits, misses, and false alarms on the 2-back task were provided after the task. Individual working memory performance was expressed as *d′* (d-prime) and calculated as *z*-scores of hits minus *z*-scores of false alarms (Stanislaw and Todorov, 1999).

#### Results and Discussion

The main goal of this experiment was to replicate the findings in Experiment 2a with an altered design and a new group of 19 participants. We measured their Stroop interference scores and the total percentage of the time during which the MIB target was subjectively invisible. As in Experiment 2a, a typical Stroop effect was observed as median reaction times for neutral (*M* = 522 ms, *SD* = 59 ms) and incongruent trials (*M* = 503 ms, *SD* = 57 ms) differed significantly [*t*(18) = 2.75, *p* = 0.013]. Accuracy scores showed a similar interference pattern [incongruent: 87% ± 7%, neutral: 93% ± 3%, *t*(18) = 3.97, *p* < 0.001, Cohen’s *d*_z_ = 0.91, Cohen’s *d*_rm_ = 1.08]. More importantly, the Stroop interference scores based on reaction times and MIB strength correlated across participants (**Figure 3B**, *right;* Spearman–Brown-adjusted split-half reliability of Stroop interference scores: 0.59; MIB duration: 0.99). To examine the specificity of this finding and in particular the possibility that the strong correlation reflected the influence of fatigue or executive control, we additionally measured the participants’ working-memory performance with a 2-back working memory task. The data show that *d′* during this task (hits: *M* = 60%, *SD* = 17%; false alarms: *M* = 18%, *SD* = 13%) neither correlated with Stroop interference [*r*(17) = -0.20, *p* = 0.41] nor with MIB strength [*r*(17) = 0.09, *p* = 0.73]. The partial correlation between Stroop interference and MIB strength while controlling for working-memory performance was essentially unchanged [*r*(17) = 0.63, *p* < 0.01]. These results suggest that individual differences in Stroop interference can be explained for about 40% by variance in MIB, independent of the experimental design, sample population, fatigue, and executive control.

It is remarkable that Stroop interference was highly correlated with MIB, but showed no correlation with n-back task performance, which is typically regarded as a measure of executive control. These findings are especially striking given the brief duration of the Stroop task (∼5 min). The involvement of executive control in the task is thought to be strongest when the task is novel; increasing time-on-task reduces a task’s effectiveness in capturing executive functions (Rabbitt, 1997). So even though the Stroop-task duration must have optimized the contribution of executive control, individual differences in Stroop interference could be predicted from individuals’ MIB scores and not from their performance on another task often used to assess executive control.

The overall performance on the 2-back task was relatively poor as compared to typical performance on the 2-back task. This may be related to the fact that we asked participants to memorize colors rather than digits or numbers –the standard stimuli in the n-back task – which may have increased task difficulty.

## General Discussion

In two separate experiments we find that individual differences in Stroop task interference relate to the relative timing of the electrophysiological P3 component for separately presented word and color stimuli (Experiment 1) and the lateral inhibition of feature representations in a perceptual illusion (Experiment 2). Specifically, Experiment 1 showed that relatively late P3 onsets to the isolated presentation of colorless words as compared to colored rectangle presentations predicted strong interference in a subsequent Stroop task. In Experiment 2 we demonstrated that people with strong Stroop interference were more likely to experience strong perceptual inhibition in the illusion MIB. These findings imply that Stroop interference measures two rather basic aspects of visual processing: (1) when an individual’s word processing is relatively slow, and (2) when an individual’s visual processing system is characterized by stronger lateral inhibition, this person is also likely to experience stronger Stroop interference.

A considerable part of the Stroop interference in our experiments was explained by word versus color P3 latency and lateral inhibition. It is important to note that we do not exclude the possibility that the remaining portion of unexplained variance in Stroop interference scores depends on executive control. We merely suggest that the strong link between the passive P3 components of the ERP and inhibitory MIB task indicates a larger role of pre-set visual processing parameters than previously assumed.

The role of processing speed of features in the Stroop task needs explanation as its significance has been questioned in Stroop-task literature (Dunbar and MacLeod, 1984; MacLeod and MacDonald, 2000). Although previous studies have demonstrated relatively weak effects of word readability on Stroop interference (Dunbar and MacLeod, 1984; MacLeod and MacDonald, 2000), proficiency in reading is a factor that explains variance in Stroop interference across individuals in a heterogeneous group of children (Protopapas et al., 2007). As the proficiency of lexical processing is reflected in P3 latencies (Taylor, 1988; Taylor and Keenan, 1990), we find it tempting to suggest that a large portion of individual variation in Stroop interference in our data is due to variations in the speed of processing words. This also suggests that P3 latency is a sensitive measure that detects small variation in word processing speed and Stroop interference in homogeneous groups (i.e., highly educated, young adults). Thus, our interpretation of this finding is to some extent related to the largely discredited ideas by Dyer (1973) that appointed a large role to the relative processing speed of colors versus words. Standardized verbal Stroop tasks, as typically used in clinics on patients, attempt to take into account the role of a patient’s word reading speed by incorporating items of colorless words that have to be read out aloud during the test (Golden, 1978). However, such control conditions have been criticized for their accuracy (Lansbergen et al., 2007a) and do not take into account our observation that roughly half of the variance in Stroop task performance is related to the relative speed between word reading and color processing.

### Explaining Individual Differences in Interference by other Factors than Executive Control

Our finding that a considerable proportion of individual variability in Stroop interference is linked to basic visual processing parameters questions the previously assumed central role of executive, top–down control in determining an individual’s interference score. The correlational approach in our experiments enabled us to circumvent potential effects of executive control. For example, the correlation between interference and MIB is unlikely to be the result of an executive process because MIB is thought to reflect lateral inhibition in the visual domain rather than top-down executive control. MIB is governed by an imbalance in the distribution of attention across competing visual features (Bonneh et al., 2001; Schölvinck and Rees, 2009) and there are no known indications that executive control underlies the illusion. As such, it is plausible that a considerable portion of the effect sizes in Stroop interference, as predicted by the MIB illusion, is governed by an early, visual mechanism that is distinct from high-order cognitive processes such as top-down executive control.

The suggestion that early mechanisms underlie Stroop interference may seem unexpected given that Stroop interference predominantly has neural correlates in non-sensory areas such as the frontal lobe and anterior cingulate cortex (e.g., Perret, 1974) – brain areas believed to be important for executive control and conflict resolution (e.g., MacLeod and MacDonald, 2000; Botvinick et al., 2001; di Pellegrino et al., 2007). On the other hand, illusory disappearances in MIB are explained by activity in the visual cortex (Donner et al., 2008, 2013; Libedinsky et al., 2009; Schölvinck and Rees, 2010). In line with proposed models that pinpoint the importance of the distinct processing pathways of words versus colors (Cohen et al., 1990; MacLeod, 1991; MacLeod and MacDonald, 2000; Roelofs, 2003), we suggest that the relative speeds of sensory processing and lateral inhibition between feature representations play crucial roles in inducing Stroop interference.

Does executive control have any influence on Stroop interference? Correlational studies suggest that executive control may have limited influence because individual scores on Stroop interference correlate weakly with performance on other executive control tasks (Miyake et al., 2000; Ward et al., 2001). In other words, Stroop-like tasks that differ in stimulus design (and thus differ in sensory processing) seem unrelated and this challenges the common assumption that they all rely on a single mechanism, namely executive control. This is in line with other recent demonstrations that empirical phenomena that were previously interpreted as a result of executive control, turn out to reflect (in part) simple cognitive mechanisms (Jacoby et al., 2003; Mayr et al., 2003; Schneider and Logan, 2005). Similarly, the present data suggest that the Stroop task does not primarily measure executive control.

### Limitations of the Present Study

The amount of interference in the Stroop task relates to performance on other tasks such as block design, digit symbol, similarities, digit span, and serial subtraction task – all kinds of tests that are typically included in intelligence tests (Shum et al., 1990; Graf et al., 1995). It is currently unclear whether intelligence underlies the correlations between Stroop interference, P3 latencies for word and color stimuli, and MIB. As we did not incorporate an intelligence test in the design, the quantification of this and perhaps reading skills should be considered in the design of follow-up experiments. Such a test may additionally provide evidence that P3 latency is selectively sensitive to the speed of word reading.

Because Experiments 1 and 2 involved different groups of participants, we do not know to what extent the portion of explained variance in Stroop interference by relative P3 latency in Experiment 1 (∼50%) overlaps with the portion of variance explained by MIB in Experiment 2 (∼40%). Alternative to the possibility that the contributions of these factors add up to 90% of explained variance in total, the factors of processing speed and lateral inhibition may mutually depend on each other, and together account for only half of the variance in Stroop interference. To test this, future correlational studies will have to incorporate an all-encompassing design in which the same participants are tested on the Stroop task, P3 latencies for isolated features, and MIB. In such studies other executive function tasks should also be added as control tasks because the current study only used the *n*-back working memory task.

### Passive Viewing versus Active Response Conflicts

A fundamental assumption underlying our approach is that passive viewing of words and colored objects (Experiment 1) and reporting the subjective visibility of the target (Experiment 2) require little or no executive control. However, one may argue that in both tasks participants had to adhere to the task instructions and pay attention to the stimuli on the display, and that these mental acts require executive control. Perhaps individuals with smaller Stroop interference also pay more attention to the word stimulus in these relatively passive tasks. Could this explain the observed correlations, and rescue the notion that individual differences in Stroop interference primarily reflect differences in executive control? We believe this is unlikely. Although P3 latency often scales with the amount of attention paid to the stimulus (Verleger, 1997), it is unclear why increased attention in the passive viewing task should affect the *difference* in the color and word P3 latencies—the variable that correlated with Stroop interference in Experiment 1. Furthermore, there is substantial evidence that increased attention to the target (Geng et al., 2007; Carter et al., 2008; Devyatko, 2011) and to maintaining steady fixation (Bonneh et al., 2010) should increase target disappearances in the MIB paradigm. This suggests that individuals with strong executive control should exhibit increased MIB and weak Stroop interference. However, we find the opposite pattern in Experiments 2a and 2b: increased MIB in participants with strong interference. Furthermore, the observation that Stroop interference and MIB illusion strength did not correlate with working memory performance in Experiment 2b makes it even less likely that individual differences in executive control underlie the link between the Stroop task and MIB task. In sum, it is most logical to conclude that pre-set dynamics in stimulus processing and lateral inhibition rather than executive control underlie the correlations reported here.

## Author Contributions

Authors MN and AV developed the research questions. Authors MN and AV designed the studies. Authors MN, AV, and SB carried out the studies and author MN analyzed the data. Authors MN and SN wrote the article and all other authors provided detailed comments on the initial versions of the article.

## Conflict of Interest Statement

The authors declare that the research was conducted in the absence of any commercial or financial relationships that could be construed as a potential conflict of interest.
