# Supplementary material for: Speed and Lateral Inhibition of Stimulus Processing Contribute to Individual Differences in Stroop-Task Performance
Source: Front Psychol. 2016 Jun 1;7:822. doi: 10.3389/fpsyg.2016.00822 (PMC4887505; doi:10.3389/fpsyg.2016.00822)
Supplement: Supplementary file 1 [file Table_1.DOCX]

# Supplemental Materials

**Table 1. Statistics concerning the correlations between the Stroop effect and the difference in P3 latency between color and word stimuli per electrode.**

| **Electrode** | **Correlation** | **P-value (*n* = 23)** |
| --- | --- | --- |
| 'O2' | -0,27 | 0,204 |
| 'F1' | 0,28 | 0,204 |
| 'Fz' | -0,07 | 0,754 |
| 'F2' | 0,02 | 0,916 |
| 'O1' | -0,11 | 0,631 |
| 'Cz' | 0,07 | 0,756 |
| 'Oz' | -0,06 | 0,786 |
| 'PO8' | 0,01 | 0,969 |
| 'TP8' | 0,11 | 0,626 |
| 'CP6' | -0,55 | 0,007 |
| 'CP4' | -0,66 | 0,001 |
| 'CP2' | -0,73 | 0,000 |
| 'CPz' | -0,25 | 0,248 |
| 'CP1' | -0,14 | 0,537 |
| 'CP3' | -0,42 | 0,045 |
| 'CP5' | -0,16 | 0,479 |
| 'PO7' | -0,25 | 0,255 |
| 'TP7' | -0,35 | 0,107 |
| 'P9' | 0,01 | 0,957 |
| 'P7' | -0,27 | 0,216 |
| 'P5' | -0,30 | 0,163 |
| 'P3' | -0,44 | 0,036 |
| 'P1' | -0,46 | 0,028 |
| 'Pz' | -0,44 | 0,034 |
| 'P8' | -0,05 | 0,837 |
| 'P6' | -0,06 | 0,780 |
| 'P4' | -0,07 | 0,755 |
| 'P2' | -0,67 | 0,000 |
| 'PO4' | -0,32 | 0,141 |
| 'POz' | -0,41 | 0,052 |
| 'PO3' | -0,45 | 0,032 |
| 'P10' | 0,23 | 0,286 |
